# *Notes from the Field*: Multistate Outbreak of *Escherichia coli* O26 Infections Linked to Raw Flour — United States, 2019

**DOI:** 10.15585/mmwr.mm7016a4

**Published:** 2021-04-23

**Authors:** Michael Vasser, Jonathan Barkley, Adam Miller, Ellen Gee, Katherine Purcell, Morgan N. Schroeder, Colin Basler, Karen P. Neil

**Affiliations:** ^1^Division of Foodborne, Waterborne, and Environmental Diseases, CDC; ^2^Oak Ridge Institute for Science and Education, Oak Ridge, Tennessee; ^3^Rhode Island Department of Health; ^4^Division of Preparedness, Response, Infectious Disease, and Emergency Medical Services, Center for Acute Infectious Disease Epidemiology, Providence, Rhode Island; ^5^Division of State Laboratories and Medical Examiners, Center for Biological Sciences, Providence, Rhode Island; ^6^Coordinated Outbreak Response and Evaluation Network, Food and Drug Administration, College Park, Maryland; ^7^New York State Department of Health, Albany, New York.

On February 20, 2019, PulseNet, the molecular subtyping network for foodborne disease surveillance, identified six Shiga toxin–producing *Escherichia coli* (STEC) O26:H11 infections with the same pulsed-field gel electrophoresis (PFGE) pattern combination. This PFGE pattern combination matched that of infections from a July 2018 outbreak that was associated with ground beef. In response, CDC initiated an investigation with federal, state, and local partners to identify the outbreak source and implement prevention measures.

CDC defined a case as STEC O26 infection with an isolate matching the outbreak strain by PFGE or related by core genome multilocus sequence typing scheme (cgMLST), with dates of illness onset during December 11, 2018–May 21, 2019. Investigators initially hypothesized that ground beef was the outbreak cause because of the PFGE match to the July 2018 outbreak and because in early interviews, patients commonly reported eating ground beef and leafy greens. Investigators used cgMLST to compare the genetic sequences of isolates from both outbreaks and determined that they fell into separate genetic clades (differing by 6–11 alleles), suggesting that something other than ground beef caused the illness in 2019. CDC noted that one patient consumed raw cookie dough and that most patients were young adult females, similar to demographic distributions of past flour-associated STEC outbreaks (*1*–*3*). Investigators developed a supplemental questionnaire focusing on beef, leafy greens, and flour exposures.

Twenty-one cases were reported from nine states (Figure). The median age of patients was 24 years (range = 7–86 years); 71% were female. Three patients were hospitalized, and none died. Among 13 patients asked about flour exposures, six reported eating, licking, or tasting raw homemade dough or batter during the week before illness onset. Three patients reported eating raw dough or batter made with the same grocery store brand of all-purpose flour, including a patient who reported eating raw dough at a bakery in Rhode Island. Overall, of 18 patients with store information, 11 reported shopping at this same grocery store chain.

**FIGURE:**
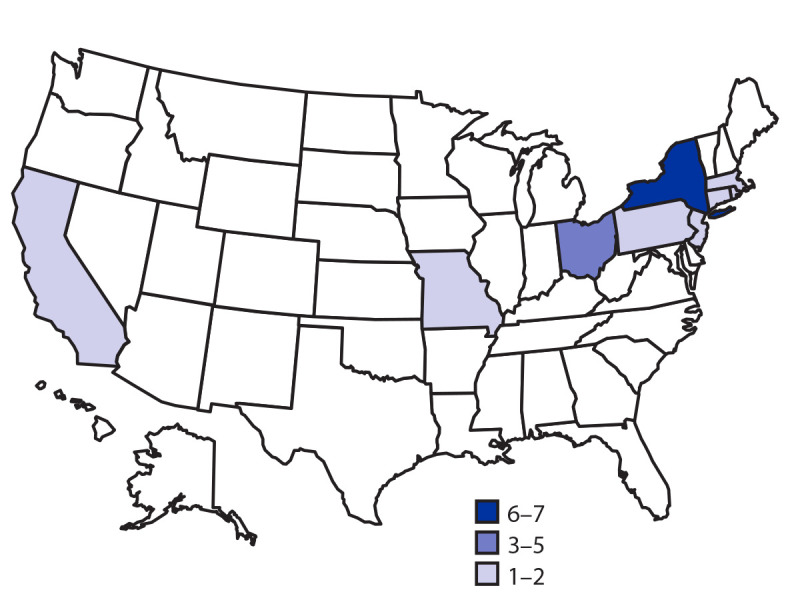
Number of patients* (N = 21) infected with the outbreak strain of *Escherichia coli* O26, by state of residence — United States, December 2018–May 2019 * California, one; Connecticut, one; Massachusetts, two; Missouri, one; New Jersey, one; New York, seven; Ohio, five; Pennsylvania, two; Rhode Island, one.

The Rhode Island Department of Health visited the bakery reported by the patient and collected flour for testing. On May 21, 2019, testing identified STEC O26 from an intact bag of all-purpose flour, which was the same grocery store brand reported by other patients. PulseNet confirmed that the STEC O26 isolated from the flour was highly related to clinical isolates using cgMLST (0–1 alleles). Product distribution records collected by the Food and Drug Administration indicated that the store brand flour purchased by six patients in three states was produced in a single milling facility in Buffalo, New York. Based on results of the investigation, the store chain recalled all lots of product from its retail locations in 11 states. The milling company also recalled all lots of this product and several other lots of flour produced in that facility, resulting in the recall of additional brands and products distributed to multiple states.

Flour is increasingly recognized as a cause of STEC outbreaks (*1*–*5*). Raw flour is not a ready-to-eat product, and this outbreak highlights the continuing risk for illness associated with consumption of flour and raw dough or batter. The investigation was aided by considering demographic information early in the investigation because these characteristics were similar to those in past flour-associated outbreaks (*1*–*3*). These similarities, coupled with the discriminatory power of cgMLST, helped to guide the consideration of alternative hypotheses regarding the outbreak source and the successful identification of flour as the cause of this outbreak.
